# Supplementary material for: Phylogenetic and biogeographical traits predict unrecognized hosts of zoonotic leishmaniasis
Source: PLoS Negl Trop Dis. 2023 May 31;17(5):e0010879. doi: 10.1371/journal.pntd.0010879 (PMC10231829; doi:10.1371/journal.pntd.0010879)
Supplement: S1 Text — We ran the initial search on February 12, 2021. We ran additional searches that included the species names of zoonotic Leishmania on February 22, 2021. Our host database was then updated in June 2021 after the publication of (1). (DOCX) [file pntd.0010879.s001.docx]

**S1 Text. Search methods for recording *Leishmania* host status:** We used the search string listed below to run search Web of Science of *Leishmania* hosts. We ran the initial search on February 12, 2021. We ran additional searches that included the species names of zoonotic *Leishmania* on February 22, 2021. Our host database was then updated in June 2021 after the publication of (1).

Search string:

AB=(("leishmania*" OR "uta" OR "chiclero ulcer" OR "pian bois") AND ("PCR" OR "sero*" OR "serum" OR "infected" OR "detected" OR "RT-PCR" OR "qPCR" OR "PCR" OR "chain reaction" OR "xenodiagnosis" OR "culture" OR "isolat*" OR "reservoir") AND ("Central America" OR "South America" OR "Latin America" OR "America*" OR "Antigua" OR "Barbuda" OR "Aruba" OR "Bahamas" OR "Barbados" OR "Grand Cayman" OR "Cayman Islands" OR "Little Cayman" OR "Cayman Brac" OR "Cuba" OR "Dominica" OR "Dominican Republic" OR "Grenada" OR "Guadeloupe" OR "Haiti" OR "Jamaica" OR "Martinique" OR "Puerto Rico" OR "Saint Barthelemy" OR "Saint Kitts" OR "Nevis" OR "Saint Vincent" OR "Grenadines" OR "Canouan" OR "Bequia" OR "Trinidad" OR "Tobago" OR "Virgin Islands" OR "Belize" OR "Costa Rica" OR "El Salvador" OR "Guatemala" OR "Honduras" OR "Mexico" OR "Nicaragua" OR "Panama" OR "Argentina" OR "Bolivia" OR "Brazil" OR "Chile" OR "Colombia" OR "Ecuador" OR "Guyana" OR "Paraguay" OR "Peru" OR "Suriname" OR "Uruguay" OR "Venezuela") AND ("animal" OR "vertebrate" OR "host" OR "wildlife" OR "livestock" OR "domestic") )

**References**

1. Azami-Conesa I, Gómez-Muñoz MT, Martínez-Díaz RA. A Systematic Review (1990–2021) of Wild Animals Infected with Zoonotic Leishmania. Microorganisms. 2021 May 20;9(5):1101.
