# Supplementary material for: Phylogenetic and biogeographical traits predict unrecognized hosts of zoonotic leishmaniasis
Source: PLoS Negl Trop Dis. 2023 May 31;17(5):e0010879. doi: 10.1371/journal.pntd.0010879 (PMC10231829; doi:10.1371/journal.pntd.0010879)
Supplement: S1 Table — (DOCX) [file pntd.0010879.s002.docx]

**S1 Table.** Final traits and sources of traits used in the analysis.

| ***trait*** | ***definition*** | ***source*** |
| --- | --- | --- |
| **neonate body mass (g)** | mass of neonates | panTHERIA |
| **adult forearm length (mm)** | total length from elbow to wrist | panTHERIA |
| **gestation length (d)** | length of time of non-inactive fetal growth | panTHERIA |
| **litter size** | average # of offspring in a litter | panTHERIA |
| **litters per year** | average # of litters per year | panTHERIA |
| **max longevity (m)** | maximum adult age | panTHERIA |
| **social group size (n)** | # of individuals in a group | panTHERIA |
| **weaning age (d)** | age when primary nutritional dependency on the mother ends and independent foraging begins to make a major contribution to the offspring’s energy | panTHERIA |
| **activity cycle** | activity cycle (nocturnal, crepuscular, diurnal) | panTHERIA |
| **dominate diet** | primary food group consumed by the animal, including groups related to sandfly habitat and exposure (insectivore, frugivore, granivore, folivore) | mammalDiet |
| **diet breadth** | number of dietary categories eaten by each species | panTHERIA |
| **home range size (km^2^)** | size of the area within which everyday activities of individuals or groups (of any type) are typically restricted | panTHERIA |
| **population density (n/km^2^)** | population size per km^2^ | panTHERIA |
| **foraging strata** | assignment to one of five foraging stratum categories (ground level, scansorial, arboreal, aerial) | EltonTraits |
| **trophic strata** | assignment to one of three trophic levels: carnivore, omnivore, herbivore | EltonTraits |
| **temperature seasonality** |  | CHELSA |
| **temperature in the warmest quarter** |  | CHELSA |
| **precipitation in the driest quarter** |  | CHELSA |
| **humidity range** |  | CHELSA |
| **climate moisture index range** |  | CHELSA |
| **main habitat** | Main habitat defined by IUCN, includes forest, savanna, shrubland, grassland, wetland, rocky area, desert, artificial | IUCN |
| **habitat breadth** | number of IUCN defined main habitats |  |
| **percent crop cover** | average percent of the range covered by crop land from 2015-2019 | IUCN, gee^a^: COPERNICUS/Landcover/100m/Proba-V-C3/Global |
| **percent tree cover** | average percent of the range covered by trees from 2015-2019 | IUCN, gee^a^: COPERNICUS/Landcover/100m/Proba-V-C3/Global |
| **percent urban cover** | average percent of the range covered by urban landscape from 2015-2019 | IUCN, gee^a^: COPERNICUS/Landcover/100m/Proba-V-C3/Global |
| **global human modification index** | aggregate measure of the human modification; mean gHm within an animal's range in 2016 | IUCN, gee^a^: CSP/HM/GlobalHumanModification |
| **range area (km^2^)** |  | panTHERIA |
| **maximum longitude of range (dd)** |  | panTHERIA |
| **minimum longitude of range (dd)** |  | panTHERIA |
| **median latitude of range (dd)** |  | panTHERIA |
| **phylogenetic distance** | Pairwise divergence time, averaged across all available sources for species pairs, reduced into five dimensions via PCoA | TimeTree |
| **invasive** | invasive in Mexico, Central America, or South America | GISD |
| **zoonotic host** | reservoir host of any zoonotic pathogen | GIDEON |
| **citation count** | number of citations found on pubmed | pubmed |
| ^a^ google earth engine | | |
