# Supplementary material for: Phylogenetic and biogeographical traits predict unrecognized hosts of zoonotic leishmaniasis
Source: PLoS Negl Trop Dis. 2023 May 31;17(5):e0010879. doi: 10.1371/journal.pntd.0010879 (PMC10231829; doi:10.1371/journal.pntd.0010879)
Supplement: S2 Table — (DOCX) [file pntd.0010879.s003.docx]

**S2 Table.** Summed Shapley Scores per species for newly predicted species, averages across 100 model iterations (0.05 percentile - 0.95 percentile). Grey boxes represent animals that are already known hosts for the subgenera.

| **Binomial** | **Order** | ***L. (Viannia)* scores** | ***L. (Leishmania)* scores** | **Reference** |
| --- | --- | --- | --- | --- |
| *Odocoileus virginianus* | Artiodactyla | 1.40 (0.01-2.58) | 1.46 (0.54-2.17) |  |
| *Pecari tajacu* | Artiodactyla | -- | 2.28 (1.84-2.83) | (1) |
| *Sus scrofa* | Artiodactyla | -- | 1.49 (0.38-2.34) |  |
| *Tayassu pecari* | Artiodactyla | -- | 2.22 (1.70-2.92) |  |
| *Bassariscus astutus* | Carnivora | -- | 0.74 (0.31-1.75) |  |
| *Canis latrans* | Carnivora | -- | 1.39 (0.75-1.92) |  |
| *Canis lupus* | Carnivora | 0.86 (0.18-1.45) | 1.84 (1.02-2.73) |  |
| *Herpailurus yagouaroundi* | Carnivora | 0.86 (0.24-1.57) | 2.20 (1.57-2.82) |  |
| *Leopardus wiedii* | Carnivora | 0.89 (0.26-1.61) | 2.74 (2.22-3.14) |  |
| *Lontra longicaudis* | Carnivora | -- | 1.74 (1.05-2.35) |  |
| *Mustela frenata* | Carnivora | -- | 1.64 (0.88-2.44) |  |
| *Nasua narica* | Carnivora | 1.29 (0.10-2.30) | 1.48 (0.70-2.19) |  |
| *Potos flavus* | Carnivora | 1.77 (1.02-2.37) | -- | (2) |
| *Procyon cancrivorus* | Carnivora | -- | 2.34 (1.57-3.01) |  |
| *Procyon lotor* | Carnivora | 1.02 (0.19-2.25) | 1.95 (1.21-2.65) |  |
| *Puma concolor* | Carnivora | 0.89 (0.23-1.69) | -- | (3) |
| *Vulpes vulpes* | Carnivora | -- | 1.91 (1.31-2.63) |  |
| *Anoura caudifer* | Chiroptera | -- | 1.49 (0.43-2.23) |  |
| *Anoura geoffroyi* | Chiroptera | 1.23 (0.21-2.31) | 2.15 (1.48-2.86) |  |
| *Artibeus phaeotis* | Chiroptera | 1.05 (0.54-1.73) | 0.97 (0.27-1.45) |  |
| *Carollia perspicillata* | Chiroptera | 2.16 (1.43-2.87) | -- | (4) |
| *Chiroderma villosum* | Chiroptera | -- | 0.98 (0.22-1.63) |  |
| *Choeronycteris mexicana* | Chiroptera | 0.60 (0.10-1.29) | -- |  |
| *Chrotopterus auritus* | Chiroptera | -- | 1.75 (0.57-2.55) |  |
| *Diclidurus albus* | Chiroptera | 1.11 (0.37-1.94) | 1.43 (0.45-2.34) |  |
| *Diphylla ecaudata* | Chiroptera | -- | 1.79 (0.82-2.42) |  |
| *Eptesicus brasiliensis* | Chiroptera | -- | 0.95 (0.23-1.73) |  |
| *Eptesicus furinalis* | Chiroptera | 1.01 (0.09-1.87) | -- |  |
| *Eptesicus fuscus* | Chiroptera | 1.11 (0.31-1.86) | 1.41 (0.72-1.94) |  |
| *Lasionycteris noctivagans* | Chiroptera | 1.41 (0.73-2.09) | 1.10 (0.28-1.70) |  |
| *Lasiurus borealis* | Chiroptera | 1.04 (0.21-1.99) | 1.24 (0.26-2.17) |  |
| *Lasiurus cinereus* | Chiroptera | -- | 1.33 (0.64-1.89) | (5) |
| *Lasiurus ega* | Chiroptera | -- | 2.11 (1.36-2.86) |  |
| *Lasiurus intermedius* | Chiroptera | -- | 0.84 (0.18-1.78) |  |
| *Leptonycteris yerbabuenae* | Chiroptera | 0.73 (0.07-1.38) | -- |  |
| *Macrophyllum macrophyllum* | Chiroptera | -- | 1.04 (0.59-1.64) |  |
| *Macrotus waterhousii* | Chiroptera | -- | 0.83 (0.15-1.49) |  |
| *Micronycteris buriri* | Chiroptera | -- | 0.86 (0.38-1.39) |  |
| *Mormoops megalophylla* | Chiroptera | -- | 1.16 (0.51-1.93) |  |
| *Myotis albescens* | Chiroptera | -- | 1.66 (0.56-2.38) |  |
| *Myotis riparius* | Chiroptera | -- | 0.88 (0.31-1.64) |  |
| *Natalus stramineus* | Chiroptera | -- | 1.08 (0.23-1.97) |  |
| *Noctilio albiventris* | Chiroptera | -- | 1.86 (1.12-2.54) |  |
| *Noctilio leporinus* | Chiroptera | -- | 1.21 (0.48-2.12) |  |
| *Nyctinomops aurispinosus* | Chiroptera | -- | 1.07 (0.51-1.87) |  |
| *Perimytotis subflavus* | Chiroptera | 1.41 (0.65-1.98) | 1.25 (0.08-1.88) |  |
| *Peropteryx macrotis* | Chiroptera | -- | 1.14 (0.29-1.78) |  |
| *Phyllostomus hastatus* | Chiroptera | 1.34 (0.54-2.08) | -- | (6) |
| *Pteronotus parnellii* | Chiroptera | 1.43 (0.66-2.19) | -- | (7) |
| *Pteronotus quadridens* | Chiroptera | 0.82 (0.07-1.52) | -- |  |
| *Saccopteryx bilineata* | Chiroptera | -- | 1.88 (1.08-2.51) |  |
| *Sturnira lilium* | Chiroptera | 2.19 (1.19-3.02) | -- | (8) |
| *Sturnira tildae* | Chiroptera | -- | 1.12 (0.25-2.11) |  |
| *Tadarida brasiliensis* | Chiroptera | 1.70 (0.86-2.53) | 2.33 (1.93-2.73) |  |
| *Thyroptera tricolor* | Chiroptera | -- | 1.25 (0.20-2.25) |  |
| *Trachops cirrhosus* | Chiroptera | -- | 0.77 (0.13-1.58) |  |
| *Uroderma bilobatum* | Chiroptera | 1.28 (0.31-2.06) | 1.82 (0.93-2.54) |  |
| *Dasypus novemcinctus* | Cingulata | -- | 3.05 (2.57-3.42) | (9) |
| *Euphractus sexcinctus* | Cingulata | 0.90 (0.22-1.61) | 1.98 (1.32-2.69) |  |
| *Priodontes maximus* | Cingulata | 0.82 (0.08-1.40) | -- |  |
| *Caluromys derbianus* | Didelphimorphia | 2.04 (1.06-2.85) | 1.23 (0.67-1.82) |  |
| *Caluromys philander* | Didelphimorphia | -- | 2.06 (1.33-2.68) | (10) |
| *Chironectes minimus* | Didelphimorphia | -- | 1.28 (0.37-1.99) |  |
| *Didelphis virginiana* | Didelphimorphia | 2.28 (1.52-3.21) | 1.77 (0.91-2.62) |  |
| *Gracilinanus agilis* | Didelphimorphia | -- | 1.17 (0.28-2.00) | (11) |
| *Lutreolina crassicaudata* | Didelphimorphia | -- | 1.41 (0.18-2.22) |  |
| *Marmosa murina* | Didelphimorphia | 1.51 (0.32-2.38) | -- | (10) |
| *Metachirus nudicaudatus* | Didelphimorphia | 1.67 (0.76-2.54) | -- | (10) |
| *Monodelphis brevicaudata* | Didelphimorphia | 1.53 (0.38-2.54) | -- |  |
| *Monodelphis domestica* | Didelphimorphia | 1.56 (0.55-2.57) | 1.85 (0.89-2.59) |  |
| *Thylamys karimii* | Didelphimorphia | 0.95 (0.00-1.89) | -- |  |
| *Tlacuatzin canescens* | Didelphimorphia | 1.03 (0.14-1.91) | -- |  |
| *Lepus europaeus* | Lagomorpha | -- | 1.25 (0.24-2.07) |  |
| *Oryctolagus cuniculus* | Lagomorpha | 0.95 (0.20-2.07) | 0.95 (0.18-1.66) |  |
| *Romerolagus diazi* | Lagomorpha | 0.96 (0.13-1.90) | -- |  |
| *Sylvilagus audubonii* | Lagomorpha | 1.36 (0.54-2.12) | 1.20 (0.49-2.06) |  |
| *Sylvilagus brasiliensis* | Lagomorpha | -- | 1.67 (0.63-2.29) | (7) |
| *Sylvilagus palustris* | Lagomorpha | -- | 0.79 (0.11-1.56) |  |
| *Tapirus terrestris* | Perissodactyla | -- | 1.22 (0.54-1.81) |  |
| *Bradypus variegatus* | Pilosa | -- | 1.85 (1.15-2.81) | (12) |
| *Cyclopes didactylus* | Pilosa | 2.14 (1.15-2.99) | 1.66 (0.86-2.37) |  |
| *Myrmecophaga tridactyla* | Pilosa | 1.36 (0.42-2.22) | -- | (7) |
| *Ateles geoffroyi* | Primates | 1.88 (1.00-2.61) | 0.97 (0.16-1.69) |  |
| *Callithrix jacchus* | Primates | 1.10 (0.24-1.84) | -- | (7) |
| *Cebus capucinus* | Primates | 1.52 (0.30-2.63) | -- |  |
| *Erythrocebus patas* | Primates | -- | 1.11 (0.33-2.16) |  |
| *Leontocebus fuscicollis* | Primates | 0.92 (0.14-1.67) | -- |  |
| *Macaca mulatta* | Primates | -- | 1.10 (0.31-2.09) |  |
| *Saguinus oedipus* | Primates | 1.67 (0.72-2.65) | 1.04 (0.12-2.20) |  |
| *Saimiri oerstedii* | Primates | 1.15 (0.12-2.30) | -- |  |
| *Saimiri sciureus* | Primates | 1.00 (0.38-1.68) | -- |  |
| *Sapajus apella* | Primates | -- | 0.91 (0.07-1.99) |  |
| *Calomys callosus* | Rodentia | 2.32 (1.61-3.09) | 2.15 (1.24-3.02) |  |
| *Calomys expulsus* | Rodentia | 0.84 (0.09-1.69) | -- |  |
| *Cavia aperea* | Rodentia | 1.11 (0.52-2.03) | -- | (7) |
| *Cerradomys subflavus* | Rodentia | -- | 1.40 (0.13-2.27) | (13) |
| *Cuniculusi paca* | Rodentia | -- | 2.45 (1.77-3.18) |  |
| *Dasyprocta leporina* | Rodentia | 0.87 (0.03-2.09) | 1.46 (0.57-2.22) |  |
| *Dasyprocta punctata* | Rodentia | 1.54 (0.57-2.82) | 0.73 (0.07-1.41) |  |
| *Dipodomys merriami* | Rodentia | -- | 1.68 (0.86-2.41) |  |
| *Dipodomys ordii* | Rodentia | 1.46 (0.59-2.22) | 1.45 (0.63-2.43) |  |
| *Galea spixii* | Rodentia | -- | 2.34 (1.76-2.96) |  |
| *Glaucomys volans* | Rodentia | 1.37 (0.64-2.07) | 0.77 (0.01-1.68) |  |
| *Heteromys salvini* | Rodentia | 1.53 (0.73-2.53) | 1.77 (1.02-2.45) |  |
| *Holochilus brasiliensis* | Rodentia | -- | 0.43 (0.07-0.89) |  |
| *Hydrochoerus hydrochaeris* | Rodentia | 1.74 (0.63-2.59) | -- | (7) |
| *Hylaeamys megacephalus* | Rodentia | 2.64 (1.72-3.29) | 2.44 (1.90-3.09) |  |
| *Kerodon rupestris* | Rodentia | -- | 1.30 (0.50-2.01) |  |
| *Myocastor coypus* | Rodentia | 0.78 (0.18-1.48) | 0.79 (0.22-1.45) |  |
| *Myoprocta acouchy* | Rodentia | -- | 0.94 (0.03-1.96) |  |
| *Myoprocta pratti* | Rodentia | 1.54 (0.52-2.29) | -- |  |
| *Neotoma mexicana* | Rodentia | 1.17 (0.29-1.78) | -- |  |
| *Neotomodon alstoni* | Rodentia | 1.11 (0.41-1.96) | -- |  |
| *Ochrotomys nuttalli* | Rodentia | 2.08 (1.38-2.73) | 1.66 (1.22-2.33) |  |
| *Oligoryzomys fornesi* | Rodentia | 1.21 (0.54-2.00) | -- |  |
| *Oligoryzomys fulvescens* | Rodentia | 1.63 (0.84-2.48) | 1.52 (0.90-2.25) |  |
| *Oligoryzomys nigripes* | Rodentia | 0.75 (0.13-1.36) | -- |  |
| *Oryzomys couesi* | Rodentia | 1.42 (0.72-2.24) | 1.22 (0.39-2.00) |  |
| *Oryzomys palustris* | Rodentia | 2.16 (1.40-2.83) | 1.70 (1.13-2.35) |  |
| *Otonyctomys hatti* | Rodentia | 0.83 (0.12-1.74) | -- |  |
| *Peromyscus aztecus* | Rodentia | 0.91 (0.09-2.08) | -- |  |
| *Peromyscus gossypinus* | Rodentia | 2.11 (1.75-2.65) | 1.62 (1.20-2.09) |  |
| *Proechimys guairae* | Rodentia | 1.23 (0.52-1.92) | 1.69 (0.82-2.30) |  |
| *Reithrodontomys fulvescens* | Rodentia | 1.12 (0.26-1.94) | 1.01 (0.29-1.65) |  |
| *Reithrodontomys humulis* | Rodentia | 1.79 (1.10-2.74) | 1.43 (0.74-2.38) |  |
| *Reithrodontomys megalotis* | Rodentia | 1.39 (0.65-2.03) | 0.89 (0.43-1.69) |  |
| *Rhipidomys mastacalis* | Rodentia | 1.39 (0.81-2.24) | -- | (11) |
| *Sciurus granatensis* | Rodentia | 1.43 (0.60-2.40) | -- |  |
| *Sciurus variegatoides* | Rodentia | -- | 0.93 (0.14-1.76) |  |
| *Sciurus vulgaris* | Rodentia | 1.48 (0.46-2.25) | -- | (2) |
| *Scotinomys teguina* | Rodentia | 0.94 (0.29-1.92) | -- |  |
| *Spermophilus variegatus* | Rodentia | 0.91 (0.16-1.84) | -- |  |
| *Tylomys nudicaudus* | Rodentia | 1.58 (0.50-2.29) | 0.84 (0.12-1.67) |  |
| *Zygodontomys brevicauda* | Rodentia | 1.87 (1.16-2.68) | 1.07 (0.27-1.84) |  |
| *Cryptotis parva* | Soricomorpha | 1.40 (0.50-2.51) | 0.99 (0.31-1.59) |  |
| *Scalopus aquaticus* | Soricomorpha | 1.78 (1.19-2.46) | 1.64 (0.84-2.25) |  |

**References**

1. Rojas-Jaimes JE, Correa-Nuñez GH, Rojas N, Cáceres-Rey O. Detection of *Leishmania (V) guyanensis* in Rhipicephalus (Boophilus) microplus (Acari: Ixodidae) collected from Pecari tajacu. Biomed Rev Inst Nac Salud. 2017 Mar 29;37(0):208–14.

2. Mimori T, Grimaldi G, Kreutzer RD, Gomez EA, McMahon-Pratt D, Tesh RB, et al. Identification, using isoenzyme electrophoresis and monoclonal antibodies, of *Leishmania* isolated from humans and wild animals of Ecuador. Am J Trop Med Hyg. 1989 Feb 1;40(2):154–8.

3. Dahroug MAA, Almeida ABPF, Sousa VRF, Dutra V, Turbino NCMR, Nakazato L, et al. *Leishmania (Leishmania) chagasi* in captive wild felids in Brazil. Trans R Soc Trop Med Hyg. 2010 Jan 1;104(1):73–4.

4. De Lima H, Rodríguez N, Barrios MA, Avila A, Cañizales I, Gutiérrez S. Isolation and molecular identification of *Leishmania chagasi* from a bat (Carollia perspicillata) in northeastern Venezuela. Mem Inst Oswaldo Cruz. 2008 Jun;103(4):412–4.

5. Castro LS, Dorval MEC, Matheus LMD, Bednaski AV, Facco GG, Silveira M, et al. *Leishmania* presence in bats in areas endemic for leishmaniasis in central-west Brazil. Int J Parasitol Parasites Wildl. 2020 Apr 1;11:261–7.

6. Gómez-Hernández C, Bento EC, Rezende-Oliveira K, Nascentes G a. N, Barbosa CG, Batista LR, et al. *Leishmania* infection in bats from a non-endemic region of Leishmaniasis in Brazil. Parasitology. 2017 Dec;144(14):1980–6.

7. Azami-Conesa I, Gómez-Muñoz MT, Martínez-Díaz RA. A Systematic Review (1990–2021) of Wild Animals Infected with Zoonotic *Leishmania*. Microorganisms. 2021 May 20;9(5):1101.

8. Savani ESMM, de Almeida MF, de Oliveira Camargo MCG, D’Auria SRN, Silva MMS, de Oliveira ML, et al. Detection of *Leishmania (Leishmania) amazonensis* and *Leishmania (Leishmania) infantum chagasi* in Brazilian bats. Vet Parasitol. 2010 Feb 26;168(1):5–10.

9. Lainson R, Shaw JJ, Ward RD, Ready PD, Naiff RD. Leishmaniasis in Brazil: XIII. Isolation of *Leishmania* from armadillos (*Dasypus novemcinctus*), and observations on the epidemiology of cutaneous leishmaniasis in north Pará State. Trans R Soc Trop Med Hyg. 1979 Jan 1;73(2):239–42.

10. Araujo Carreira JC, Magalhães M de AFM, Brazil RP, da Silva AVM. *Leishmania* in Marsupials—An Overview of Infection Records in the Americas and Australia. Open J Anim Sci. 2017;07(03):315–43.

11. Quaresma PF, Rêgo FD, Botelho HA, da Silva SR, Moura AJ Júnior, Neto RGT, et al. Wild, synanthropic and domestic hosts of *Leishmania* in an endemic area of cutaneous leishmaniasis in Minas Gerais State, Brazil. Trans R Soc Trop Med Hyg. 2011 Oct 1;105(10):579–85.

12. Muñoz-García CI, Sánchez-Montes S, Villanueva-García C, Romero-Callejas E, Díaz-López HM, Gordillo-Chávez EJ, et al. The role of sloths and anteaters as *Leishmania spp.* reservoirs: a review and a newly described natural infection of *Leishmania mexicana* in the northern anteater. Parasitol Res. 2019 Apr;118(4):1095–101.

13. Tonelli GB, Tanure A, Rego FD, Carvalho GM de L, Stumpp R, Ássimos GR, et al. *Leishmania (Viannia)* *braziliensis* infection in wild small mammals in ecotourism area of Brazil. PLoS ONE. 2017 Dec 28;12(12):e0190315.
