# Supplementary material for: Phylogenetic and biogeographical traits predict unrecognized hosts of zoonotic leishmaniasis
Source: PLoS Negl Trop Dis. 2023 May 31;17(5):e0010879. doi: 10.1371/journal.pntd.0010879 (PMC10231829; doi:10.1371/journal.pntd.0010879)
Supplement: S3 Table — (DOCX) [file pntd.0010879.s004.docx]

### **S3 Table.** Complementary evidence of predicted hosts. Complementary evidence is defined as published literature that suggests animals may be exposed to and/or infected by zoonotic *Leishmania*, but was not robust enough to include the animal in the analysis as a known host. P = predicted by our model; K = known host (the animal has been naturally infected by a *Leishmania* species known to cause leishmaniasis in humans.

| **Binomial** | **Common name** | ***L. (V)* host** | ***L. (L) host*** | **complementary evidence** | **References** |
| --- | --- | --- | --- | --- | --- |
| *Calomys callosus* | Large vesper mouse | P | P | experimental infection | (1) |
| *Galea spixii* | Spix's yellow-toothed cavy | -- | P |  | (2) |
| *Tadarida brasiliensis* | Mexican free-tailed bat | P | P |  | (3) |
| *Didelphis virginiana* | Virginia opossum | P | P | experimental infection; significant overlap w/ *L. mexicana* vectors | (4–6) |
| *Vulpes vulpes* | Red fox | -- | P | *L. infantum* host in Europe | (7) |
| *Lepus europaeus* | European hare | -- | P |  | (8) |
| *Oryctolagus cuniculus* | European rabbit | P | P |  | (9) |
| *Sus scrofa* | Wild boar | -- | P | *Leishmania* infection or exposure but not confirmed as zoonotic | (10) |
| *Canis latrans* | Coyote | -- | P |  | (11) |
| *Lontra longicaudis* | Neotropical otter | -- | P |  | (12) |
| *Procyon cancrivorus* | Crab-eating racoon | -- | P |  | (13) |
| *Monodelphis domestica* | Gray short-tailed opossum | P | P |  | (4) |
| *Euphractus sexcinctus* | Six-banded armadillo | P | P |  | (14) |
| *Cavia aperea* | Brazilian guinea pig | P | K |  | (15) |
| *Hydrochoerus hydrochaeris* | Capybara | P | K |  | (15) |
| *Sciurus granatensis* | Red-tailed squirrel | P | -- |  | (12) |
| *Caluromys derbianus* | Derby's wooly opossum | P | P | *Leishmania* infection but not confirmed as zoonotic; vector feeds on animal | (4) |
| *Procyon lotor* | Common racoon | P | P | significant overlap w/ *L. mexicana* vectors | (5,6) |
| *Chiroderma villosum* | Hairy big-eyed bat | -- | P |  |  |
| *Eptesicus brasiliensis* | Brazilian brown bat | -- | P |  |  |
| *Saccopteryx bilineata* | Greater sac-winged bat | -- | P |  |  |
| *Uroderma bilobatum* | Tent-making bat | P | P |  |  |
| *Cuniculus paca* | Lowland paca | K | P |  |  |
| *Neotoma mexicana* | Mexican woodrat | P | -- |  |  |
| *Oligoryzomys fulvescens* | Fulvous pygmy rice rat | P | P |  |  |
| *Oryzomys nigripes* |  | P | -- |  |  |
| *Oryzomys couesi* | Coues's rice rat | P | P |  |  |
| *Reithrodontomys fulvescens* | Fulvous harvest mouse | P | P |  |  |
| *Potos flavus* | Kinkajou | P | K |  |  |
| *Nasua narica* | White-nosed coati | P | P |  |  |
| *Leopardus wiedii* | Margay | P | P |  |  |
| *Dasyprocta leporina* | Red-rumped agouti | P | P | vector feeds on animal | (16) |

**References**

1. Rosa RB, Costa MS da, Teixeira SC, Castro EF de, Dantas WM, Ferro EAV, et al. *Calomys callosus*: An Experimental Animal Model Applied to Parasitic Diseases Investigations of Public Health Concern. Pathog Basel Switz. 2022 Mar 17;11(3):369.

2. Barbosa PBBM, de Queiroz PVS, Jerônimo SMB, Ximenes M de FF de M. Experimental infection parameters in *Galea spixii* (Rodentia: Caviidae) with *Leishmania infantum* *chagasi*. Mem Inst Oswaldo Cruz. 2008 Sep;103(6):545–8.

3. Berzunza-Cruz M, Rodríguez-Moreno Á, Gutiérrez-Granados G, González-Salazar C, Stephens CR, Hidalgo-Mihart M, et al. *Leishmania (L.) mexicana* Infected Bats in Mexico: Novel Potential Reservoirs. PLoS Negl Trop Dis. 2015 Jan 28;9(1):e0003438.

4. Araujo Carreira JC, Magalhães M de AFM, Brazil RP, da Silva AVM. Leishmania in Marsupials—An Overview of Infection Records in the Americas and Australia. Open J Anim Sci. 2017;07(03):315–43.

5. Stephens CR, González-Salazar C, Sánchez-Cordero V, Becker I, Rebollar-Tellez E, Rodríguez-Moreno Á, et al. Can You Judge a Disease Host by the Company It Keeps? Predicting Disease Hosts and Their Relative Importance: A Case Study for Leishmaniasis. PLoS Negl Trop Dis. 2016 Oct 7;10(10):e0005004.

6. Stephens CR, Heau JG, González C, Ibarra-Cerdeña CN, Sánchez-Cordero V, González-Salazar C. Using biotic interaction networks for prediction in biodiversity and emerging diseases. PloS One. 2009 May 28;4(5):e5725.

7. Karayiannis S, Ntais P, Messaritakis I, Tsirigotakis N, Dokianakis E, Antoniou M. Detection of *Leishmania infantum* in red foxes (*Vulpes vulpes*) in Central Greece. Parasitology. 2015 Nov;142(13):1574–8.

8. Tsokana CN, Sokos C, Giannakopoulos A, Mamuris Z, Birtsas P, Papaspyropoulos K, et al. First evidence of *Leishmania infection* in European brown hare (*Lepus europaeus*) in Greece: GIS analysis and phylogenetic position within the *Leishmania spp*. Parasitol Res. 2016 Jan;115(1):313–21.

9. García N, Moreno I, Alvarez J, de la Cruz ML, Navarro A, Pérez-Sancho M, et al. Evidence of *Leishmania infantum* Infection in Rabbits (*Oryctolagus cuniculus*) in a Natural Area in Madrid, Spain. BioMed Res Int. 2014;2014:318254.

10. Brazil RP, Desterro MD, Nascimento SB, Macau RP. Natural infection of a pig (*Sus scrofa*) by *Leishmania* in a recent focus of cutaneous leishmaniasis on the Island of São Luis, Maranhão. Mem Inst Oswaldo Cruz. 1987 Mar;82(1):145.

11. Rosypal AC, Alexander A, Byrd D, Weaver M, Stewart R, Gerhold R, et al. Survey Of Antibodies To *Leishmania spp.* In Wild Canids From Pennsylvania And Tennessee. J Zoo Wildl Med. 2013;44(4):1131–3.

12. Azami-Conesa I, Gómez-Muñoz MT, Martínez-Díaz RA. A Systematic Review (1990–2021) of Wild Animals Infected with Zoonotic *Leishmania*. Microorganisms. 2021 May 20;9(5):1101.

13. Richini-Pereira VB, Marson PM, Hayasaka EY, Victoria C, da Silva RC, Langoni H. Molecular detection of *Leishmania spp*. in road-killed wild mammals in the Central Western area of the State of São Paulo, Brazil. J Venom Anim Toxins Trop Dis. 2014 Jun 16;20:27.

14. de Oliveira Barbosa W, Coelho TG, da Costa TO, Paiz LM, Fornazari F, Langoni H, et al. Antibodies to *Toxoplasma gondii*, *Leishmania spp.,* and *Leptospira spp*. in Free-Ranging Six-Banded Armadillos (*Euphractus sexcinctus*) from Northeastern Brazil. J Wildl Dis. 2020 Apr;56(2):486–8.

15. Caldart ET, Pinto-Ferreira F, Matos AMRN de, Pascoal ATP, Bertão-Santos A, Mitsuka-Breganó R, et al. Evaluation of an active and early surveillance methodology for visceral leishmaniasis by molecular detection in road-killed wild fauna. Rev Bras Parasitol Veterinária. 2021;30(2):e027920.

16. Kocher A, de Thoisy B, Catzeflis F, Valière S, Bañuls AL, Murienne J. iDNA screening: Disease vectors as vertebrate samplers. Mol Ecol. 2017;26(22):6478–86.
