# Supplementary material for: Phylogenetic and biogeographical traits predict unrecognized hosts of zoonotic leishmaniasis
Source: PLoS Negl Trop Dis. 2023 May 31;17(5):e0010879. doi: 10.1371/journal.pntd.0010879 (PMC10231829; doi:10.1371/journal.pntd.0010879)
Supplement: S1 Fig — (DOCX) [file pntd.0010879.s005.docx]

###
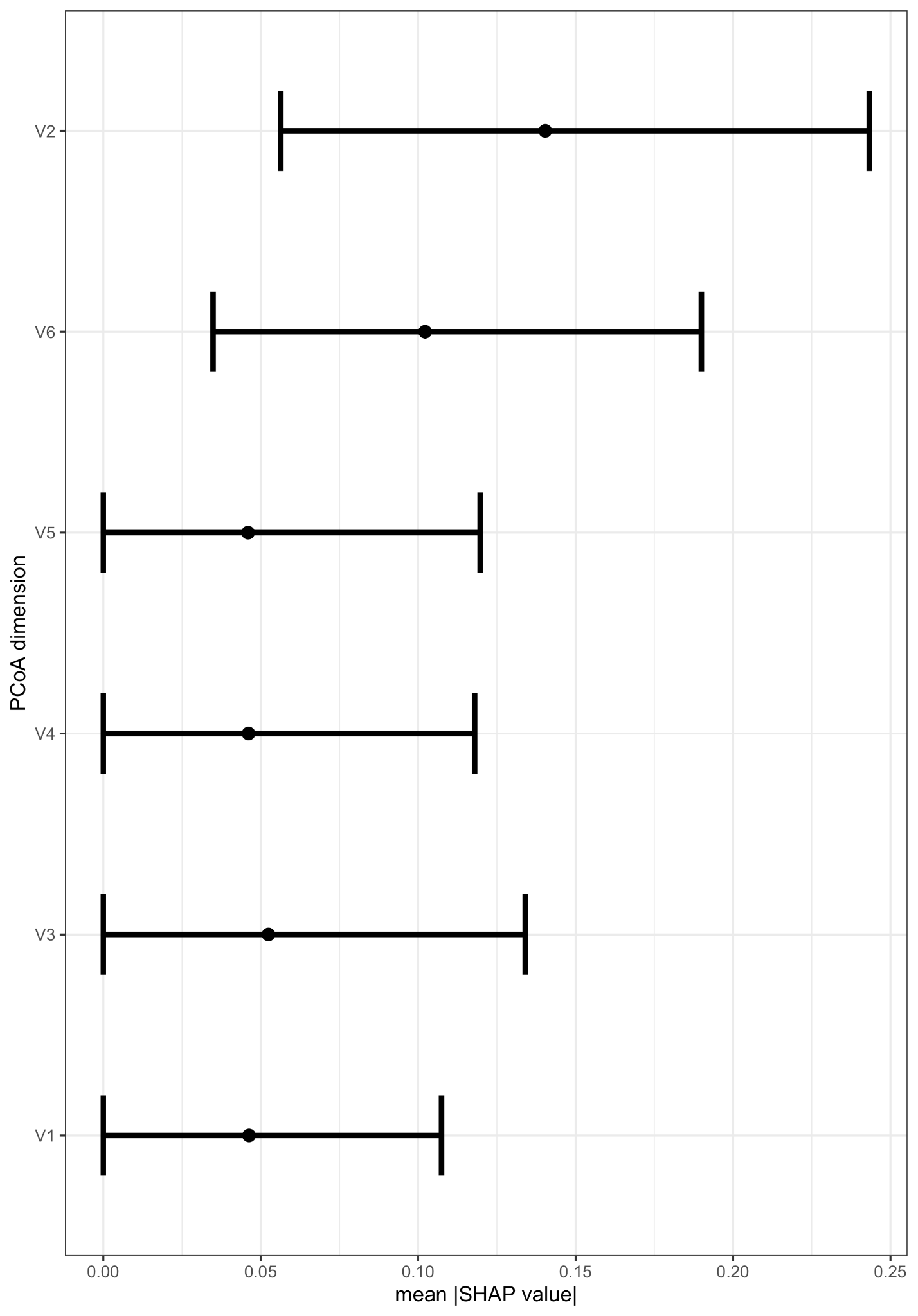


**S1 Fig. Importance of each PCoA phylogenetic dimension in predicting *L. (Viannia)* host status.** Points are the mean Shapley importance for the PCoA dimension across all mammals, bars represent the 0.05-0.95 percentiles.
