# Supplementary material for: Phylogenetic and biogeographical traits predict unrecognized hosts of zoonotic leishmaniasis
Source: PLoS Negl Trop Dis. 2023 May 31;17(5):e0010879. doi: 10.1371/journal.pntd.0010879 (PMC10231829; doi:10.1371/journal.pntd.0010879)
Supplement: S2 Fig — (DOCX) [file pntd.0010879.s006.docx]

**
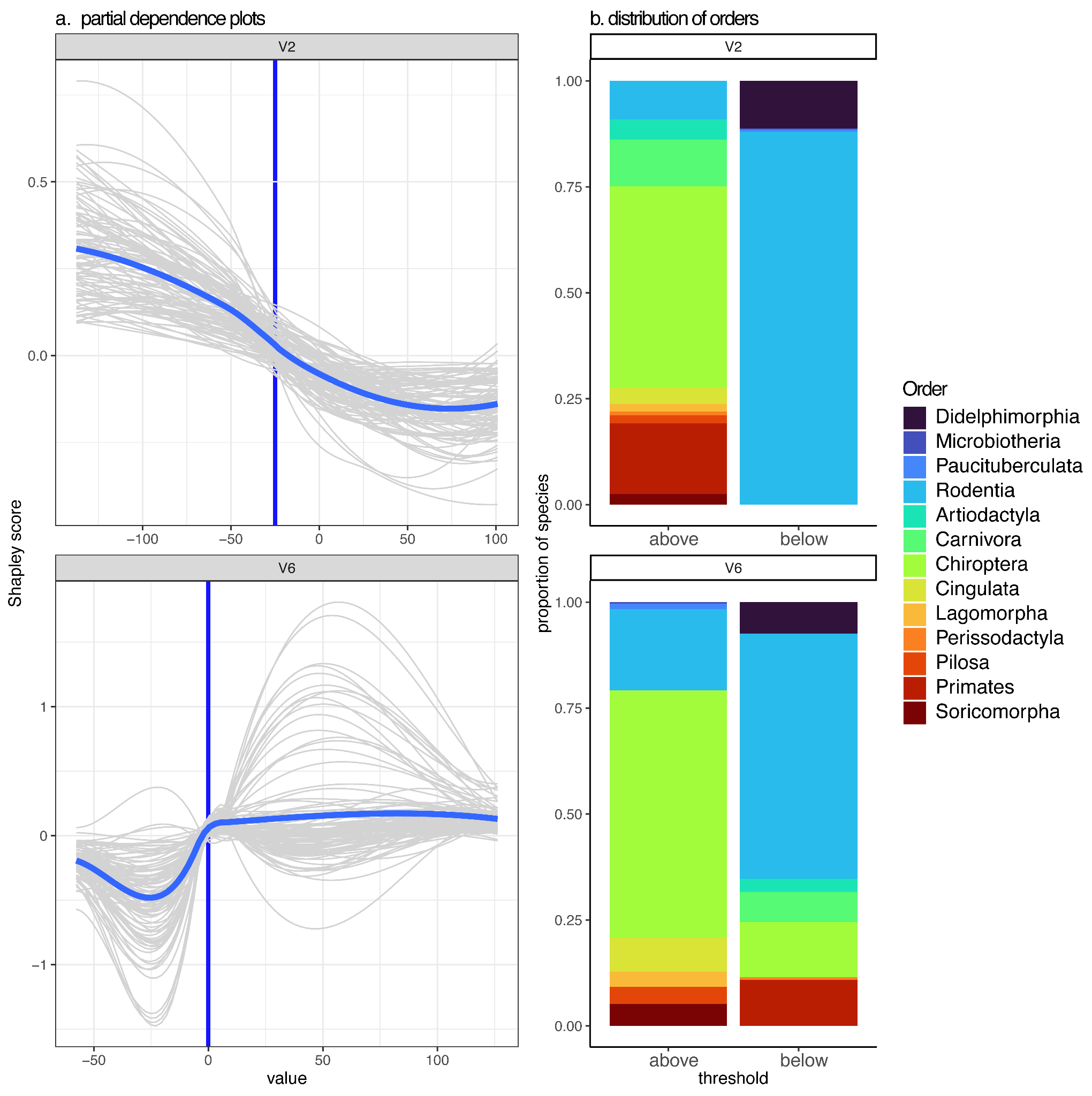
**

**S2 Fig. (a)** Shapley partial dependence plots showing the effect of PCoA dimensions on *L.* (*Viannia*) host status. Blue non-linear lines represent the average effect across model iterations , while grey lines show each individual model iteration (model fit with 70% of data). Dark blue vertical line represents a threshold value, where the dimension switches from having a negative effect on host status to a positive effect on host status. Only dimensions with importance scores > 0 for > 95% of model iterations are shown. **(b)** distribution of hosts within each order with PCoA dimension values above or below the threshold.
