# Supplementary material for: Phylogenetic and biogeographical traits predict unrecognized hosts of zoonotic leishmaniasis
Source: PLoS Negl Trop Dis. 2023 May 31;17(5):e0010879. doi: 10.1371/journal.pntd.0010879 (PMC10231829; doi:10.1371/journal.pntd.0010879)
Supplement: S3 Fig — (DOCX) [file pntd.0010879.s007.docx]

**
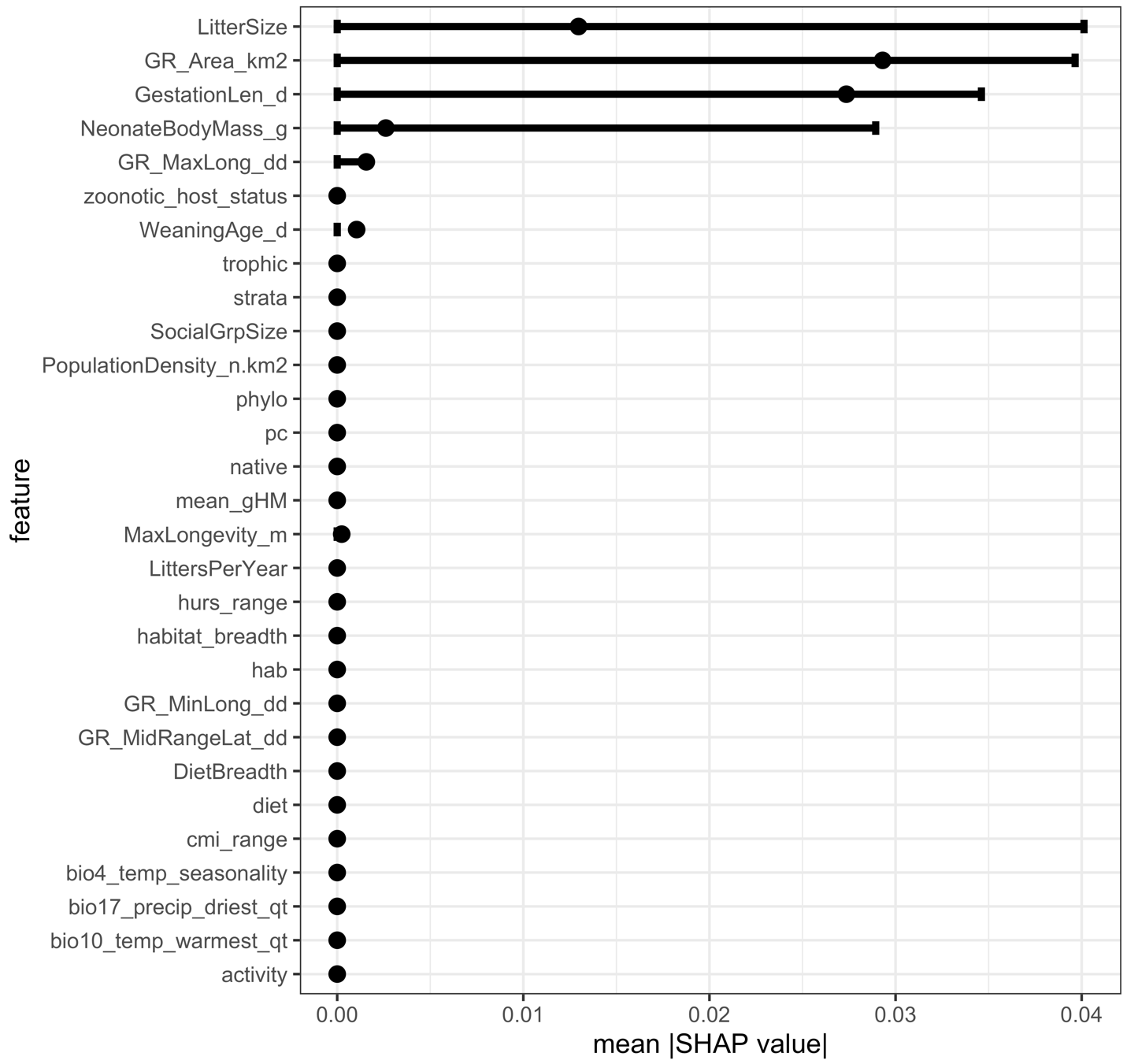
**

**S3 Fig. Features inconsistently contribute to predictions for study effort of mammals included in the *L. (Viannia)* model.** Global feature contribution for traits predicting study effort of mammals included in the *L. (Viannia)* model Points are the absolute value of the mean Shapley importance (mean |SHAP value|) for the trait across all mammals, bars represent the absolute values of the 0.05-0.95 percentiles. No trait consistently contributes to model predictions across all model bootstrapping iterations (i.e., there is no trait where the 0.05 percentile is greater than 0). Further, traits with average values > 0 do not correspond to traits that consistently predict *L. (Viannia)* host status.
