# Supplementary material for: Phylogenetic and biogeographical traits predict unrecognized hosts of zoonotic leishmaniasis
Source: PLoS Negl Trop Dis. 2023 May 31;17(5):e0010879. doi: 10.1371/journal.pntd.0010879 (PMC10231829; doi:10.1371/journal.pntd.0010879)
Supplement: S6 Fig — (DOCX) [file pntd.0010879.s010.docx]

**
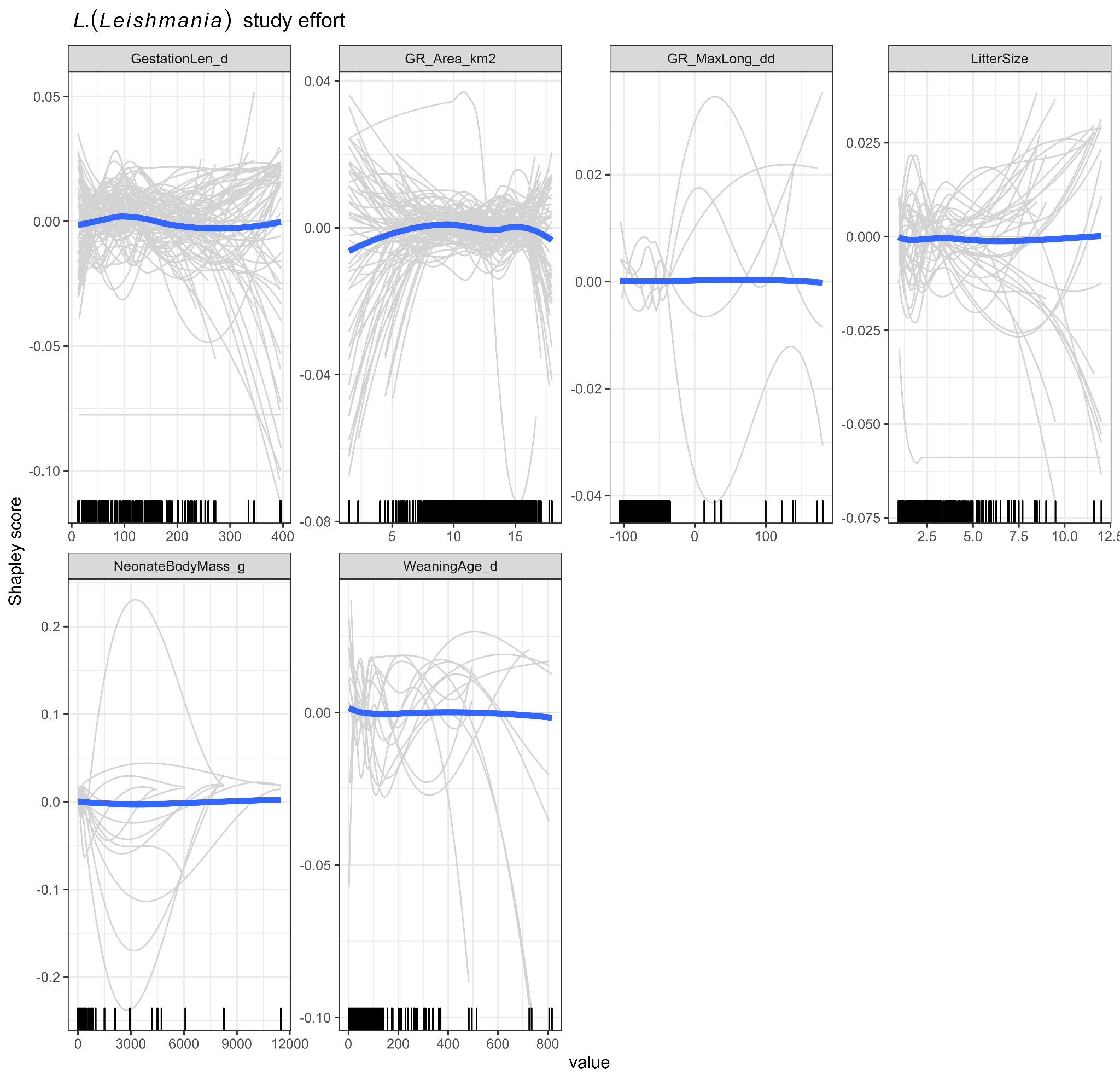
**

**S6 Fig. Response of study effort predictions to mammal traits are inconsistent and do not mirror functional forms of response of *L. (Leishmania)* host status to mammal traits.** Shapley partial dependence plots showing the effect of mammal traits on study effort of mammals included in the *L. (Leishmania)* model. Blue non-linear lines represent the average effect across model iterations, while grey lines show each individual model iteration (model fit with 70% of data). Functional form varies greatly across model iterations. Only traits where the absolute value of average contribution was > 0 are shown. Rug plots on the x-axis indicate the distribution of the data.
