# Supplementary material for: Phylogenetic and biogeographical traits predict unrecognized hosts of zoonotic leishmaniasis
Source: PLoS Negl Trop Dis. 2023 May 31;17(5):e0010879. doi: 10.1371/journal.pntd.0010879 (PMC10231829; doi:10.1371/journal.pntd.0010879)
Supplement: Attachment — Submitted filename: Glidden_LeishHost_ReviewerComments_jan18.docx [file pntd.0010879.s011.docx]

**Reviewer 1:**

***Methods***

It is worth mentioning that I do not work directly with the modeling approach employed by authors and, therefore, I was not able to judge more fully the pertinence of the analyzes carried out. The authors presented the analyzes and explanations for the use and validation of each one of them, which seemed accurate to me. I have two comments to be considered by authors:

Lines 170-173: Authors attributed the same category (0) for animals that were examined and negative for Leishmania infection and for those that was never surveyed. Wouldn't it be better to divide this analysis: the negatives from those that represent a sample void?

This is a good point. In an ideal world, we would have both true positives (1s) and true negatives (0s), as well as species of unknown status. However, because sampling is so sporadic and logistically challenging, we do not have enough true negatives and true positives to differentiate them. As a result, we use the model to differentiate true positives (1s) from non-positives (i.e., animals that either have been surveyed and were negative or have not been surveyed). This allows us to identify which species have not yet been tested but have a high probability to be a host, given the trait profiles of the known hosts.

Lines 172-173: Authors include in the analysis infection determined by serology. But this technique does not differentiate infections by Leishmania (Leishmania) from Leishmania (Viannia) due to cross-infections. How did the authors deal with this in the separate analysis of the two subgenres?

We initially ran the analysis with all zoonotic *Leishmania* (found in the Americas) grouped together but later updated the paper to split the two groups. When we split the groups, we removed species that were only identified via serology because, as you stated, serological tests do not adequately distinguish between *Leishmania* species and subgenera. We removed serology from the text.

*Line 207-209: Infection was determined via detection of Leishmania species specific antigens or genetic material. Species identification was performed either using animal tissue samples directly or using live parasites cultured from animal tissue.*

***Results***

Figure 3: Put the "Trait Type" caption outside the frame of L. (Viannia) because it serves for the 2 frames. Or put it in both frames

Updated.

Supplementary Table 2: Add an extra column with the bibliography that defines the gray boxes

Added a reference column and references list.

***Conclusions***

Authors were very careful and responsible with the conclusions that were presented in the manuscript, clearly presenting and discussing the study limitations

Thank you!

***Editorial & Data Presentation Modifications***

The authors focused on the still poorly knowledge on wild hosts of zoonotic Leishmania species to predict the unknown hosts using machine learning approach. They used trait-profiles of known hosts to identify potentially unknown hosts, especially concerning biogeography, phylogenetic distance, and study effort. Besides the above-mentioned aspects, I have also these comments:

1)      Authors should not include their figures in the Introduction. This section should present the state-of-art of the study theme, including previous (and not the current) studies. Figure 1, for example, does not represent human cases, as is cited in line 64. This will lead authors to also revise the figure numbering, because figures should be numbered in the order of appearance in the text.

Thank you for this suggestion. We additionally have clarified that the legend is not representing case numbers.

2)      Introduction (line 88): I suggest considering as "may act as competent hosts". The course of an infection is dependent on factors related to the parasite population, the host, environmental factors, health status and concomitant infections, among others. This means that these 60 species of mammals have already been demonstrated to be able of acting as a reservoir, being source of vector infection, but this characteristic is not permanent, and these animals will not always be able to establish a course of infection that will lead to the transmission of the parasites, i.e., that will lead them to act as a reservoir. Only field studies can define if a putative reservoir is acting as reservoir in a given time-space scale.

We agree and have included rephrased “are competent hosts” to “may act as competent hosts” as well as “become infectious..” to “can become infectious…”.

*Lines 103-105: …* *of which around 60 may act as competent hosts (i.e., animals can become infectious once infected, and may maintain the pathogen in the environment as reservoir hosts)…*

3)      Oryzomys megacephalus is a homotypic synonym of Hylaeamys megacephalus, which is the correct name.

We have updated *Oryzomys megacephalus* to *Hylaeamys megacephalus (Lines 342, 380, supplementary materials table 2).* We used *Oryzomys* in the text as this is the nomenclature used in the Pantheria database. Along with *Oryzomys megacephalus,* we changed species names in the manuscript back to nomenclature used by IUCN (all changes were made in supplemental table 2 and 3: *Herpailurus yagouaroundi, Perimytos subflavus, Leontocebus fuscicollis, Sapajus apella, Cuniculus paca, Heteromys salvini.*

4)      Discussion (line 516): Wouldn't Thrichomys laurentius also be part of this group of competent hosts (doi:10.1371/journal.pntd.0000589) ?

*Thrichomys laurentius* has been found to be infected with zoonotic *Leishmania* in the wild and so was included in our analysis as a known potential host. The group of competent hosts described in this paragraph refers to newly predicted hosts that have not been found to be infected in the wild (or ever tested for *Leishmania* infection in the wild) but have been found to become infectious in laboratory experiments.

We added a couple of sentences to clarify this point in the text:

*Line 589-596: Host competence (i.e., the ability to become infected and infectious) has been demonstrated in the lab for four of the predicted species: the Virginia oppossum (Didlephis virginiana), the large vesper mouse (Calomys callosus), spix’s yellow toothed cavy (Galea spixii), and the Mexican free-tailed bat (Tadarida brasiliensis) (69,73–75). Although host competence for these animals has been tested in the laboratory, infection/exposure has not been tested in the wild or animals have not tested positive in the wild (possibly due to minimal sampling of the species). Our model suggests that these animals are also likely to be naturally infected in the wild and thus warrant further investigation.*

5)      There are some references cited in number and other in text. Please revise this throughout the manuscript to fit them in the journal’s format.

Thank you for identifying this issue. We have updated the manuscript with the correct reference format.

**Reviewer 2:**

***Methods***

Yes, the methods are adequate.

***Results***

The results are correct, well ordered and clear. I think there is an excess of supplementary material.

Thank you! While there is a lot of supplementary material, we feel it is necessary to include all of the materials to ensure that our study is repeatable and that the results are robustly supported.

***Conclusions***

The discussion is very exhaustive, comparing well the data obtained in this study with the literature. In addition, the usefulness of this prediction is well explained.

Thank you!

***Editorial & Data Presentation Modifications***

The subgenus is called Leishmania (Viannia), not Vianna. Please correct this throughout the text.

Thank you for identifying this issue. We have fixed the spelling throughout the text.

Line 84: Please specify order Diptera, family Psychodidae

We updated the line to include taxonomic information (*Line 77*).

Line 84: It would be more correct to speak of potential hosts, as the fact that an animal may be susceptible to infection alone cannot be considered a true host. Please, add “potential” before host.

Updated to include potential (*Line 93*).

Line 122: It is not correct to speak of opportunistic sampling in the study of zoonotic Leishmania, as in most cases targeted sampling of the animal populations under study is performed. Please, remove the word “opportunistic”.

After further consideration, we felt as though this sentence was misleading and removed it from the text. The paragraph now starts with “Study designs optimal for detecting…” (*Line 113*).

Line 335: Please, add the degree symbol (˚)

Added.

Line 354: In figure 2, specify L. (Viannia) and L. (Leishmania) and write them in italics in the figure of the subgenera (c).

We italicized the subgenera in Figure 2c.

Line 376: Please, add the degree symbol (˚)

Added.

Lines 409 and 410: Specify the numbers of predicted hosts in each order.

Updated to specify the number of predicted hosts in each order. The text now reads as:

*Line 476-478: …predicted hosts for both subgenera fell within the Rodentia order (L. (Leishmania) N = 27; L. (Viannia) N: = 34), with a similar number of predicted Leishmania (Leishmania) hosts within the Chiroptera order (N = 32).*

Line 418: The numbering of the references is missing

Updated.

Line 440: The numbering of the references is missing

Updated.

Line 469: Remove “e.g” when talking about references.

Removed “e.g” in front of references throughout document.

Line 475: Italics and capital letter in “Leishmania”

Updated.

Lines 499 and 500: The numbering of the references is missing

Updated.

Lines 510 and 511: The numbering of the references is missing

Updated.

Line 514: The numbering of the references is missing

Updated.

Lines 518 and 519: The numbering of the references is missing

Updated.

Line 544: Put the reference at the end of the sentence, not at the beginning.

Updated.

Lines 545 and 546: The numbering of the references is missing

Updated.

The italics are absent in the names of genera and species throughout the reference section.

Updated.

Supplementary table 3: review the italics and references numbering.

Updated italics and references.

**Summary and General Comments**

It is a truly novel and comprehensive study. Perhaps the methods part is too dense, but it is well explained.

Thank you!

I think it is a good prediction tool, but it would be interesting to compare the prediction with the reality, especially in those areas where, according to the data obtained, the distribution of Leishmania is extended.

We agree and have expanded upon this point in the Discussion.

*Line 619-622: Next steps should include ground truthing the role of newly identified potential hosts in the zoonotic Leishmania transmission cycle, through field sampling and laboratory experiments, through engagement with local communities…*

*Line 637-640: Testing these hosts for leishmaniasis infection beyond the current putative boundaries of zoonotic Leishmania ranges would help to evaluate if range expansion has already occurred as well as provide an updated baseline for future monitoring.*

In addition, it can be very useful on a smaller scale, in possible outbreaks of human leishmaniasis that may occur, to look for implicated hosts.

We agree and have included this point in the Discussion.

*Line 625-627: For instance, host predictions may provide a starting point for implication of hosts during local outbreaks of human leishmaniasis by providing a targeted list of likely suspects to sample.*

**Reviewer 3:**

This is an interesting article that uses machine learning models to identify host taxa involved in Leishmania transmission. This work is important because it provides insight on the potential role of understudied wildlife species in Leishmania transmission. The approaches used are novel and well explained. Below is a list of minor comments/suggestions, mostly focused on needing to strengthen the introduction which reads a bit vague currently, and points of clarification needed in the methods and discussion. One important thing to highlight a bit more is the meaning of these findings for surveillance purposes. Since the predictions are based on infection data with no information on reservoir status for known species for example, cautious recommendations need to be provided. Generally, I found this article compelling and I believe it should be considered for publication once edits have been made and additional information provided.  

Specific comments:
Line 49 might need define "burden" in this context. Suggest providing an estimated number to avoid being vague.

We agree and have included an estimated number of cases and disability-adjusted life years lost (DALYs) in the Americas per year.

*Line 54 -56:* *Leishmaniasis is a zoonotic, vector-borne disease of poverty with a high burden throughout the Americas: within Latin America there are an estimated 58,500 new cases per year and 54,050 years of life lost due to disability.*

Line 51-52: “In some areas, risk of infection is increasing in geographic extent.” Suggest rewording to be less vague.

We included specific geographic regions to make the wording less vague.

*Lines 58-60: Across the Americas, particularly in Central America, the southeastern United States, and perimeters of the Amazon Basin, risk of infection is increasing in geographic extent and elevation.*

Line 55-57: The summary could be improved to be more specific and focused on the research findings rather than disease background.

We improved the summary to focus on our specific objectives, methods, and outcomes.

*Line 60 – 69: While it is known that Leishmania parasites, the causative agent of leishmaniasis, are maintained in the environment via a mammalian host, the full suite of wildlife hosts has yet to be documented, which significantly hinders control efforts. Here, we use machine learning and ecological and evolutionary trait profiles of known hosts to identify unrecognized potential wildlife hosts of Leishmania. We identify 136 mammals in the Americas that are likely to be exposed to and infected by zoonotic Leishmania in the wild. The high number of unrecognized potential hosts emphasizes a need to better invest in studying the ecological epidemiology of leishmaniasis. The study provides information and tools to support targeted intervention and management of this important poverty-associated disease.*

Line 64: It’s unusual to have a figure in the introduction. Suggest moving to the methods or supplementary materials.

We have removed the figures from the Introduction.

Line 65: “Hotspots” is one word.

Updated.

Lines 69-82: The introduction is quite long. Suggest trimming the introduction starting with this paragraph as the background on the biology of the pathogen is probably not needed in so much detail, but rather highlighting specific background related to the study objectives (spatial-temporal, host phylogenetics, etc) is more important.

We removed this paragraph from the Introduction and moved it to the start of the Methods (*Line 169-181*). In addition, the following text was added to the Introduction to provide more relevant background information:

*Lines 96-101: Leishmaniasis was first diagnosed by Western medicine in the Americas in 1909, at the time dogs and foxes were believed to be the only zoonotic hosts (11). Continued research throughout the century hypothesized that rodents, and in some cases opossums and sloths, were the primary zoonotic hosts of these pathogens (11). However, especially with the advancement of molecular diagnostic techniques, the number of taxonomic diversity of hosts has since expeditiously increased.*

Line 70: Spillover is one word.

Updated.

Line 76: This needs to be referenced properly. More generally, the authors need to check all the references again as there is a lot of switching between number and Chicago style.

Thank you for identifying this issue. We have updated the references throughout using Vancouver style (the style requested by the journal).

Line 86: Can you provide examples of what these interventions would look like?

We have provided a few examples to strengthen this section of the text:

*Lines 87-94: For instance, clearing vector habitat proximate to households, supporting populations of natural enemies that regulate populations of vectors or small mammals (e.g., fish and mosquito larvae), and/or modifying distribution of wildlife food sources are sustainable and effective interventions in preventing transmission of flaviviruses, henipaviruses, Plasmodium spp. (malaria), and Brucella abortus (brucellosis) (10,11). Similar interventions could be employed against Leishmania; however, successful application necessitates species identification of vectors, hosts, and their corresponding interactions with their biotic and abiotic environment.*

Line 87: By wildlife hosts, do the authors mean wildlife species?

We do mean wildlife species and have updated the line to read:

*Line 101-102: In the Americas, there are now 137 species of wildlife that are recognized as potential hosts …*

Line 92: Same comment as above; it’s not typical to have figures in the introduction

We have removed the figures from the Introduction.

Line 107: What is meant by "broad surveillance"? As in passive surveillance? A reference is also needed here.

After further consideration, we felt as though this sentence was misleading and removed it from the text.

Line 110: Suggest changing "dedicate" to "target".

Updated.

Line 126: Suggest deleting “In brief”.

Removed “In brief.”

Line 140-141: This information may be better positioned in the methods section.

We removed the sentence from the Introduction as the specifics of extreme gradient boosted regression trees are already discussed in the Methods.

Line 148-149: Are these reservoir hosts? I saw the authors explain in the methods but suggest explaining earlier in the introduction.

These can be but are not necessarily reservoirs, we added the following text to clarify:

*Lines 172-173: …and may act as reservoirs given the density and distribution of their populations…*

Line 167: What search string was used and when was it done? Also wondering why the authors use Web of Science here but PubMED for citation numbers?

We added the search string as a supplementary text 1.

We first conducted a search to find all hosts using Web of Science as it searches through multiple datasets. As leishmaniasis is of medical importance, nearly all studies identifying infections in wildlife were on the PubMed database. We searched PubMed for citation number as this database contained the vast majority of studies describing sampling wildlife for *Leishmania* infection and the R package *easyPubMed* made tallying of study effort highly efficient and repeatable.

Line 172-173: If the authors are using serology data, then they can't technically be talking about infection, only exposure, since with serology data all that is known is that an individual was infected sometime in the past, and no evidence of current infection. Thus, the authors either need to remove the serology data or change the wording from "infected" to "exposed" throughout the article.

We initially ran the analysis with all zoonotic *Leishmania* (found in the Americas) grouped together but later updated the paper to split the two groups. When we split the groups, we removed species that were only identified via serology because serological tests do not adequately distinguish between *Leishmania* species and subgenera. We removed serology from the text.

Lines 178-180: Would be good to say what percent of animal taxa that included

Included:

*Lines 216-218: In total, 86.64% (1460/1685) of species were retained for the L. Viannia analysis and 87.24% (1470/1685) of species were retained for the L. Leishmania analysis.*

Line 192: Suggest defining “land-use intensity”.

We replaced land-use intensity with the specific variable associated with it (global human modification index) and briefly defined the index:

*Line 231-232: …and global human modification index, an aggregate measure of anthropogenic pressure on a landscape.*

Lines 211-221: Great explanation. It would be good to have this explanation in the context of other machine learning models (e.g., RF, SVM) for folks less familiar with these models.

We added a few lines on why the XGboost algorithm is best suited for our analysis over other machine learning methods:

*Lines 264-269: The ability to handle non-random patterns of missing data and extremely unbalanced data makes the XGBoost algorithm best suited for our study as compared to other machine learning methods, such as random forest and support machine vector, which severely overfit unbalanced data (random forest) and/or cannot handle missing data without use of imputation (random forest, support machine vector).*

Line 222: It would be good to indicate the number of traits that were removed after this step and how the authors decided which ones to include vs. leave out.

We included the requested details:

*Lines 270-274: For the L. Viannia analysis, 32/66 were retained in the final analysis; for the L. Leishmania analysis, 33/66 were retained. To maximize the number of traits retained in the model, out of a pair of correlated traits, the one with the highest number of correlations to other traits and/or the lowest data coverage was dropped.*

Line 262: Would be good to see the R packages listed somewhere. Additionally, will the authors be sharing their data and code?

We list and cite each R package that was used for analysis. The code and data are located on the first author’s github page:

*Line 351-352: The full record of species infection status and references, trait database, and R code can be found at https://github.com/ckglidden/leish_hosts.*

Line 311: I like figure 1, but wondering if the heatmap color needs to be changed as it’s a little challenging to see differences. Maybe try yellow to red or blue-yellow-red?

We updated the color scheme to make it easier to see differences within and across the maps.

Line 328: The figures in the supplementary materials showing all 30 features are quite interesting. Might be worth moving them to the main text.

The figure in the supplement is the trait importance for the model predicting the number of citations using animal traits. The purpose of it is to show that the trait profiles that contribute to study effort predictions are different than the trait profiles that contribute to host status predictions, thus demonstrating that our results for hosts are not simply showing which mammals are well-studied.

We only show a limited number of traits in Figure 3 in the text (the figure showing the contribution of each trait to host predictions) as these are the only traits that were significant after running a permutation test (i.e., the traits removed were those that were important in <5% of model iterations).

Line 355: Not totally clear why figure 2 is first reported in the introduction.

Removed figures from Introduction.

Line 407-408: Given the large number of new hosts identified, it might be worth reemphasizing that these are just hosts that could be infected (or exposed for the serology) - i.e., not predicted reservoirs.

Thank you for this suggestion. We added a definition to remind the reader that these are not necessarily reservoir hosts.

*Line 473-476: Our literature search identified 137 wild mammals in the Americas as known zoonotic Leishmania hosts, and our model predicted there to be an additional 136 likely hosts (animals that are likely to be exposed and infected in the wild, but not necessarily infectious upon infection).*

Lines 475-477: This will require a date of when the search was done.

Date added (*Line 550: (searched October 10, 2022).)*

Line 486-488: This seems a bit off topic and may derail the readers from the actual goal of the paper. It’s good to raise the issue, but suggest not dwelling on it too much.
Additionally, this paragraph reads like the last or before-last paragraph of the manuscript as it is broadening out, but then the authors dive us back into model result interpretation. So may need to move paragraphs around a bit.

We re-organized the discussion to first provide a short summary of the findings, then discuss the trait profiles of the hosts that were used to make predictions, and finally specifically discuss the predictions. We re-organized the discussion of hosts to start by interpreting the results in light of other research (i.e., discuss corroborating evidence for host predictions), then how the results can generally be used to improve surveillance, followed by a concluding paragraph with the big take-home message.

Lines 524-527: This paragraph is so short it’s not really worthy of a paragraph. Suggest expanding, merging with another paragraph, or deleting.

We combined this paragraph with the second to last paragraph, merging it with how our results can support surveillance work (see below).

Lines 540-541: So how can these results be used for surveillance? Need to provide clear and cautious guidance with these types of findings. Perhaps make a point of saying that this is "a first step".

We updated the second to last paragraph to emphasize that this research is a first step in host discovery work as well as providing an example for how this work can aid current surveillance efforts.

*Line 616 - 640: Overall, models for both subgenera identified a number of new hosts, including but not limited to the nine-banded armadillo, the margay, the large vesper mouse, and the large headed rice rat, that can be targeted for future surveillance and research to determine their ecological significance in leishmaniaisis transmission and spillover to humans. Next steps should include ground truthing the role of newly identified potential hosts in the zoonotic Leishmania transmission cycle, through field sampling and laboratory experiments, through engagement with local communities, and iteratively updating the current analysis as new information is collected, taking careful consideration to not perpetuate the effects of study bias. Although a cautious first step in host discovery work, our study can immediately inform surveillance efforts and study design. For instance, host predictions may provide a starting point for implication of hosts during local outbreaks of human leishmaniasis by providing a targeted list of likely suspects to sample. While the study of leishmaniasis has historically focused on rodents and small marsupials, our study suggests that capture methods should more commonly be supplemented by bat mist-netting and/or techniques to capture small to medium sized carnivores. Finally, many newly predicted hosts have ranges that extend throughout the southeastern USA. Ecological niche models suggest that climate warming may increase the range of Leishmania vectors as far north as Canada, and leishmaniasis endemicity within the US was recently confirmed (80–82). Similarly, the range of predicted hosts for L. (Viannia) extends farther south through Argentina than currently known hosts. Our analysis provides tools for monitoring leishmaniasis risk under global change as these hosts could be used as sentinel species for tracking leishmaniasis range expansion. Testing these hosts for leishmaniasis infection beyond the current putative boundaries of zoonotic Leishmania ranges would help to evaluate if range expansion has already occurred as well as provide an updated baseline for future monitoring.*
