# Supplementary material for: Phylogenetic and biogeographical traits predict unrecognized hosts of zoonotic leishmaniasis
Source: PLoS Negl Trop Dis. 2023 May 31;17(5):e0010879. doi: 10.1371/journal.pntd.0010879 (PMC10231829; doi:10.1371/journal.pntd.0010879)
Supplement: Attachment — Submitted filename: Response to Reviewers March 27.docx [file pntd.0010879.s012.docx]

**Response to Reviewers**

Reviewer #1: Authors satisfactorily answered the reviewers’ comments and modified the manuscript as requested. However, I suggest authors to include the answer in which they justify the use of “non-positive” instead of “true-negative” (first comment of Reviewer 1) in the methos or even discussion of the manuscript.

Thank you for this suggestion. We updated the text to include the rationale for using non-positive versus true-negative:

*Line 205-212: The latter category (non-positives) includes animals that have been tested for Leishmania and have not been found to be infected as well as those that have not been tested for Leishmania infection. Ideally, the analysis would include true-positives (1) and true negatives (0). However, due to the intensive, systematic sampling required to declare an animal as a true-negative (longitudinal sampling across large geographic scales), there is not enough data to conduct the analysis with true-negatives. Our analysis (using true-positives and non-positives) still enables us to identify species that have not yet been tested but have a high probability of being a host.*

Other minor corrections are:
1) Line 220: Exclude “from”

Updated.

2) Figure 1 is divided into a,b,c,d. But in the legend and in the text e, f, g, h are still mentioned. Review this.

Updated.

3) Line 409: Revise “there is are is high”

Updated.

4) Line 552: I suggest changing “Bolsonaro” for "federal administration from 2019-2022"

Updated.

5) Line 590: “Didelphis”, not “Didlephis”

Updated.
